# *Arthrospira platensis* as Bioremediator of Rhenium Mono- and Polymetallic Synthetic Effluents

**DOI:** 10.3390/microorganisms10112109

**Published:** 2022-10-26

**Authors:** Inga Zinicovscaia, Liliana Cepoi, Ludmila Rudi, Tatiana Chiriac, Nikita Yushin, Dmitrii Grozdov

**Affiliations:** 1 Department of Nuclear Physics, Joint Institute for Nuclear Research, 6 Joliot-Curie Str., Dubna 1419890, Russia; 2 Department of Nuclear Physics, Horia Hulubei National Institute for R&D in Physics and Nuclear Engineering, 30 Reactorului Str., P.O. Box MG-6, 077125 Bucharest, Romania; 3 Laboratory of Physical and Quantum Chemistry, Institute of Chemistry, 3, Academiei Str., MD-2028 Chisinau, Moldova; 4 Laboratory of Phycobiotechnology, Institute of Microbiology and Biotechnology, 1, Academiei Str., MD-2028 Chisinau, Moldova

**Keywords:** *Arthrospira platensis*, biochemical analysis, proteins, rhenium, molybdenum, copper, bioremediation

## Abstract

Rhenium is a scarce and highly important metal for industry and technology. In the present study, the cyanobacterium *Arthrospira platensis* (Spirulina) was used to remove rhenium and related elements (Mo and Cu) from mono- and polymetallic synthetic effluents. Metal ions in different concentrations were added to the culture medium on the first, third, and fifth days of biomass growth, and their uptake by the biomass was traced using ICP-AES technique. The accumulation of rhenium in the biomass was dependent on the chemical composition of the effluents, and the highest uptake of 161 mg/kg was achieved in the Re-Cu system. The presence of rhenium, copper, and molybdenum affected the productivity of Spirulina biomass and its biochemical composition (proteins, carbohydrates, lipids, phycobiliproteins, the content of chlorophyll *α* and *β*-carotene). With the growth of biomass in the presence of rhenium or rhenium and molybdenum, a pronounced increase in productivity and protein content was observed. The presence of copper in systems has a negative effect on biomass productivity and biochemical composition. *Arthrospira platensis* may be of interest as a bioremediator of rhenium-containing effluents of various chemical compositions.

## 1. Introduction

Rhenium (Re) is one of the rarest (7 × 10^−8^%) and most widely dispersed elements on Earth [1]. The unique physicochemical properties and extremely high melting point have predetermined the widespread use of rhenium in nuclear, aerospace, rocket, and petrochemical technologies [2,3,4]. The wide industrial application of rhenium and its limited availability compared to high demand make it one of the most expensive chemical elements [5]. In recent years, the world market price of Re has fluctuated between 6000–10,000 USD [2]. Re is traditionally obtained as a by-product of roasting and hydrometallurgical treatment of molybdenum and copper concentrates [1]. The concentration of Re in such ore bodies ranges from 100 to 2500 ppm [4]. 

Due to its strategic importance, growing demand, high cost, and scarcity of raw materials, Re extraction and recycling have attracted remarkable attention from industry and scientists [6]. This task can be solved either by improving traditional technologies for its recovery, or by developing new techniques for its recovery from wastes and secondary raw materials [2]. The current technology for Re recovery involves capturing Re_2_O_7_ from gases produced during Mo and Cu pyrometallurgical processing [4]. It can be recovered together with tungsten, molybdenum, platinum, and other metals from spent catalysts, which contain about 0.3 wt% Re [6,7]. Shen and co-authors [6] reviewed metallurgical technologies for extracting and recycling Re from primary and secondary resources, respectively. Abisheva et al. [2] summarized the technologies of rhenium recovery from mineral raw materials in Kazakhstan. Anderson et al. [8] described the primary and secondary processing technologies for rhenium extraction. 

It is known that during the process of extraction, part of Re is dispersed as volatile Re_2_O_7_ in soils, and as ReO_4_^−^ions in industrial effluents and water [3]. The main disadvantages of traditional techniques used for Re recovery include the high operating costs and energy consumption, low efficiency at a metal concentration of less than 100 mg/L, and the production of large volumes of toxic sludge, which may lead to secondary environment pollution and require additional resources for treatment [3,9]. Bioremediation is an efficient, environmentally safe, and cost-effective approach of metal removal or recovery from the environment [10]. It has been reported that microorganisms of different taxonomic groups, including bacteria, fungi, and cyanobacteria, enzymatically reduce or oxidize metal ions into less toxic forms or remove them from the environment through complexation, adsorption, ion exchange, precipitation, and coordination [11]. 

Microorganisms were not so actively applied for the removal/extraction of rhenium from solutions. In several studies, the biosorption of rhenium from aqueous solutions using microorganisms was described [3,12,13,14]. *Bacillus megaterium* [13] and *Acidithiobacillus ferrooxidans* [15] were used for rhenium bioleaching from spent refinery catalyst and molybdenite concentrate. Cyanobacteria can be considered as an optimal alternative for Re recovery from wastewater. It is known that cyanobacteria are the most numerous and diverse group of photosynthetic phylum, whose adaptive capacity, along with the ability to tolerate extreme conditions, make them ubiquitous in aquatic and terrestrial environments [16,17]. Among cyanobacteria, *Arthrospira platensis* is widely used for metal removal not only from batch systems [18,19,20], but also from real industrial effluents [21,22]. In previously performed research, Spirulina was mainly applied as biosorbent (dry biomass), whereas for bioremediation in situ studies, it is important to develop renewable metal accumulators using living biomass. Therefore, it is very important to investigate the bioaccumulation capacity of microorganisms at different stages of the technological cycle of cultivation.

The aim of the present study was to trace the bioaccumulation capacity of *Arthrospira platensis* grown in the presence of rhenium mono- and multimetal synthetic effluents added to the cultivation medium at different stages of biomass growth, as well as to evaluate the effect of metal ions on the biomass biochemical composition and antioxidant activity.

## 2. Materials and Methods

### 2.1. Chemicals

The chemicals used in the present study, NaReO_4_, CuSO_4_, and Na_2_MoO_4_ (analytical grade), were obtained from Sigma Aldrich, Darmstadt, Germany.

### 2.2. Synthetic Effluents

Four synthetic effluents with the following composition were modeled: system 1: Re-10 mg/L; system 2: Re-10 mg/L and Mo-5.0 mg/L; system 3: Re-10 mg/L and Cu-5.0 mg/L; and system 4: Re-10 mg/L, Cu-5.0 mg/L and Mo-5.0 mg/L (by metal). 

### 2.3. Bioaccumulation Experiment 

In the present study, the *Arthrospira platensis (A. platensis)* strain CNMN-CB-02 (Institute Microbiology and Biotechnology, Chisinau, Moldova) was used as an object of research. To obtain the inoculum, Spirulina was grown in a medium with the following composition (g/L): NaNO_3_—2.5; NaHCO_3_—8.0; NaCl—1.0; K_2_SO_4_—1.0; MgSO_4_∙7H_2_O—0.2; CaCl_2_—0.024; FeSO_4_·7H_2_O—0.01; H_3_BO_3_—0.00286; MnCl_2_∙4H_2_O—0.00181; CuSO_4_∙5H_2_O—0.00008; MoO_3_—0.000015; and FeEDTA-1 mg/L. For the experiment, the same medium, but without microelements (Cu and/or Mo) included in the analyzed systems, was used.

The experiment was carried out in 1 L Erlenmeyer flasks with a working volume of 700 mL, and the amount of inoculum was 0.4 g/L. To assess the effect of metal ions on Spirulina bioaccumulation capacity and biochemical composition, metal ions were added to the cultivation medium at different stages of biomass growth: lag phase (corresponds to first day of cultivation), exponential phase (corresponds to third day of cultivation), and stationary phase (corresponds to fifth day of cultivation). Spirulina was cultivated with the pH of the medium being 8–10; temperature, 28–30 °C; light intensity of 37–55 μmol photons m^−2^ s^−1^; continuous illumination; slow periodic shaking. The duration of the cultivation cycle in all cases was 6 days. On the sixth day, the biomass was separated from the cultivation medium by filtration: part of the biomass was standardized with distilled water to a concentration of 10 mg/mL and used for biochemical tests; the other part of the biomass was dried at 105 ± 2 °C and used to determine the metal content in the biomass. 

The control was biomass grown under the same conditions, but without the addition of ions of the studied metals. The biomass was cultivated in the same conditions, but without addition of studied metal ions served as control. 

### 2.4. Determination of Metal Content in Biomass

The content of Re, Cu, and Mo in Spirulina samples was determined using an inductively coupled plasma-optical emission spectrometer, PlasmaQuant 9000 Elite (Analytik Jena, Jena, Germany). Before analysis, the samples were treated with 3 mL of trace amounts of pure HNO_3_ (Sigma-Aldrich, Darmstadt, Germany) and 1 mL of H_2_O_2_ p.a. (Sigma-Aldrich, Darmstadt, Germany), and then digested at 180 °C in a Mars 6 microwave digestion system (CEM, Matthews, NC, USA). After cooling, the digested samples were quantitatively transferred into 10 mL flasks and made up to the volume with bidistilled water. Calibration solutions and standards for measurements were prepared from IV-STOCK-27 (Inorganic Ventures, Christiansburg, VA, USA) standard solution. All control standards were analyzed after every 5 samples.

### 2.5. Biochemical Analysis

The protein content in the biomass was determined by the Lowry method, and calculated from the calibration curve plotted for bovine serum albumin [23]. The calculation of the content of carbohydrates, determined using Anthracene-9(10H)-one, was done on the basis of the calibration curve plotted for glucose [24]. Lipids were determined using phosphovanillin reagent [25]. The content of phycobiliproteins in the biomass was calculated using formulas based on molar coefficients for pigments [26]. The amount of chlorophyll a and β-carotene, both determined in ethanolic extracts of the biomass, was measured at 665 nm for chlorophyll and 450 nm for β-carotene. Quantitative calculation was performed on the basis of the calibration curves.

The content of malonic dialdehyde (MDA) in the biomass was determined based on the reactive substances of thiobarbituric acid (TBA), and calculated using the extinction coefficient of the complex product of the MDA-TBA [27].

### 2.6. Antioxidant Activity

The antioxidant activity of ethanolic and water extracts was determined using the radical cation ABTS (2,2 azinobis 3-ethylbenzothiazoline-6-sulfonic acid) [28]. A detailed description of the biochemical test and determination of antioxidant activity can be found in Yushin et al.’s study [29].

### 2.7. Statistical Analysis

All experiments and measurements were performed in three repetitions. Statistical analysis was performed by one-way analysis of variance (ANOVA) using Statistica 12 (Student’s *t*-tests). 

## 3. Results and Discussion

### 3.1. Bioaccumulation Capacity of A. platensis

Bioremediation and biodegradation are the most promising tools for the decontamination of wastewater and polluted natural water. The use of Spirulina in bioremediation studies is associated with its ability to cope with high alkalinity, temperature, salt concentration, and the presence of different pollutants [29]. Cyanobacteria have developed physiological regulatory mechanisms to adapt to changes in the level of metals in the environment [30]. In the processes of bioremediation, cyanobacteria at different stages of growth interact with wastewater of different chemical compositions. Thus, the bioremediation capacity of cyanobacteria may depend on the phase of their growth, as well as on the characteristics of wastewater, such as the chemical composition and concentration of metals. 

**Figure 1:**
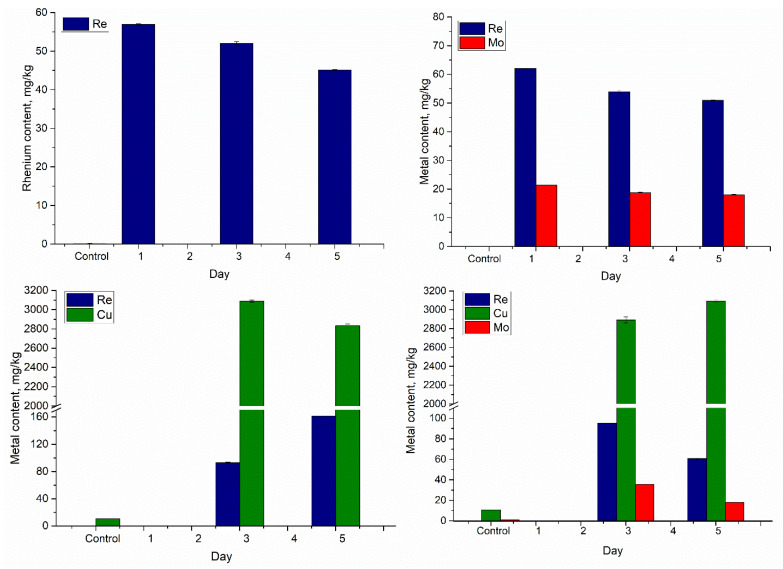
Metal uptake by Spirulina biomass from rhenium-containing effluents with different chemical composition (cultivation days 1, 3, 5, when metal ions were introduced into the cultivation medium; the metal content was determined in the Spirulina biomass grown for six days).

Since rhenium is recovered mainly from molybdenum and copper ores, four systems were modeled with the following chemical compositions: Re, Re-Mo, Re-Cu, and Re-Cu-Mo. The uptake of metal ions was traced using the ICP-OES technique, and the obtained results are presented in Figure 1. 

According to Figure 1, in the Re-system, the highest rhenium bioaccumulation of 57 mg/kg was achieved in the lag phase (first day of biomass growth). The addition of rhenium on the third day of Spirulina growth, which is a stage of exponential growth characterized by rapid cell multiplication, led to an increase in its content in the biomass by 500 times relative to the control. The lowest uptake of rhenium, 45 mg/kg, occurred when the metal was added on the fifth day of biomass growth, the stationary stage of Spirulina growth, which is marked by a pronounced resistance of the microbial culture to toxicants. 

Microorganisms, including cyanobacteria, undergo various physiological processes during growth, which can lead to a different cell metabolism and different composition of cell walls, directly affecting their ability to accumulate chemical elements [31,32,33,34]. Thus, the bacteria, *Bacillus subtilis*, during the exponential phase, removed more Cd and Fe from the solution than in the stationary phase, which was explained by the availability of a larger number of metal binding sites in the exponential phase [32]. For *Pseudomonas putida,* the difference in the number of binding sites in the exponential and stationary phases was insignificant, whereas in the death phase, the number of carboxyl groups was significantly higher than in other growth phases. Thus, the removal of U from the solution did not change significantly depending on the bacterial growth phase [31]. Chromium(VI) removal by *Ochrobactrum* sp. cells in the logarithmic phase was 1.4 times higher than in the stationary phase [34]. As already mentioned, Spirulina accumulated more rhenium during the lag phase of biomass growth. The lag phase is the initial stage in the life of cyanobacterial culture, when cells adapt to a new environment before starting exponential growth [35]. Lag phase metabolism may include the activation of signaling pathways and transcriptional changes leading to the upregulation of the protein assembly, nucleotide metabolism, lipopolysaccharide biosynthesis, respiration, and other processes that are needed for differentiation and multiplication [36]. This phase supports the formation of metabolic enzymes to facilitate the growth of bacteria and their adaptation to a stressful environment [37]. During the lag phase, due to physiological needs, many metal ions, especially major elements, are accumulated by microbial cells [35]. The active accumulation of rhenium by Spirulina in this phase can be explained by its uptake during the process of the accumulation of essential metals. 

It should be mentioned that rhenium has no biological functions, and its effect on microorganisms has been poorly studied. Slate et al. [38] showed that rhenium has an inhibitory effect on *Klebsiella pneumoniae* and *Acinetobacter baumannii* at concentrations of 13 and 11.7 mg/L, respectively. 

In the case of the Re-Mo system, the same pattern as in the Re-system was observed for both elements, and the greatest accumulation of elements was achieved with their addition on the first day of biomass growth. Thus, the content of rhenium increased 590 times compared to the control, and molybdenum—23 times. With the addition of metals in the exponential and stationary phases of biomass growth, their content in the biomass increased 515 and 488 times, respectively, for rhenium and 20 and 18 times, respectively, for molybdenum. Unlike rhenium, molybdenum is of essential importance for almost all biological systems, as it is required for enzymes that catalyze various key reactions in the global metabolism of carbon, sulfur, and nitrogen [39]. 

In the Re-Cu system, the accumulation of rhenium differed from the previously described systems. The highest uptake of metal by biomass of 161 mg/kg, which is 1540 times more than in the control, was achieved with the addition of metals in the stationary phase of biomass growth. It is important to note that in these systems, the biomass did not survive when metals were added at the lag phase of biomass growth (Figure 1). Since, in the Re and Re-Mo systems, the biomass survived at all stages of metal addition, copper can be considered as a toxic agent for Spirulina biomass. Copper ions play a dual role as a nutrient and toxicant for cyanobacteria, participating in a multitude of photochemical processes, as well as in the production of reactive oxygen species (ROS) [36], which leads to severe damage to lipids, proteins, DNA, and other cytoplasmic molecules [40]. The highest uptake of copper by biomass was achieved with the addition of metal on the third day of biomass growth (3090 mg/kg), and when the metal was added on the fifth day, the copper content in the biomass was 2830 mg/kg. The high accumulation of copper in the exponential phase may be associated with the high expression of its transporter genes [34].

In the Re-Cu-Mo system, as in the case of the Re-Cu system, Spirulina biomass did not survive when metal ions were added to the cultivation medium on the first day of biomass growth. The highest uptake of rhenium (95 mg/kg) and molybdenum (36 mg/kg) was in the exponential phase. It is suggested that the high abundance and availability of nutrients in the logarithmic phase stimulates the synthesis of cell components, which contains a large number of molecules with metal-binding sites. Copper was more actively accumulated by the biomass in the stationary phase (3090 mg/kg), which is in agreement with [35].

The mechanism of uptake of multi-metal ions by microorganisms is quite complex. The combination of metals in a multicomponent system can have both synergistic and antagonistic effects [41]. The accumulation of rhenium in the Re-Mo system was not affected by the presence of molybdenum ions. The lower rate of molybdenum accumulation in this system can be explained by (i) its lower concentration in solution in comparison with rhenium, (ii) competition of elements, and (iii) inhibition of molybdenum uptake by SO_4_^2−^ [42]. In the Re-Cu system, the accumulation of rhenium in the exponential and stationary phases was 1.8 and 3.6 times higher, respectively, than in the Re- system. Thus, the synergistic relationship between rhenium and copper was observed. In the Re-Cu-Mo system, the increase in the accumulation of rhenium in comparison with the Re system was not so noticeable. Its content increased 1.8 times at the exponential stage, and 1.3 times at the stationary stage. The lower rate of rhenium uptake can be explained by the competition of rhenium and molybdenum for binding sites. In the stationary phase, a high accumulation of copper was accompanied by a lower uptake of rhenium and molybdenum. Thus, in this case, an antagonistic effect of copper on the uptake of rhenium and molybdenum was observed.

### 3.2. Effect of Rhenium-Containing Effluents on A. platensis Productivity and Protein Content

**Figure 2:**
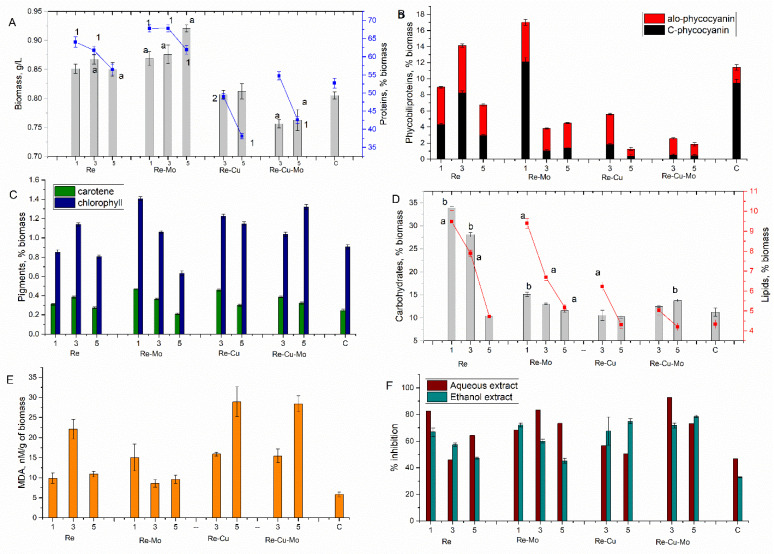
(**A**). Biomass and protein content in Spirulina biomass grown in media supplemented with metals (a—*p* < 0.005 for differences in biomass between control and experiment; 1—*p* < 0.001 for differences in protein content between control and experiment; 2—*p* < 0.01 for differences in protein content between control and experiment); (**B**) change in the content of phycobiliproteins in Spirulina biomass grown in media supplemented with metals (*p* < 0.005 for differences between control and experiment); (**C**) changes in pigment content in Spirulina biomass grown in media supplemented with metals (*p* < 0.005 for differences between control and experiment, for all parameters, except for the case of carotene in Re-Mo system at stationary stage); (**D**) changes in the content of carbohydrates and lipids in the biomass of Spirulina grown in media supplemented with metals (a—*p* < 0.005 for differences in lipid content between control and experiment; b—*p* < 0.005 for differences in the content of carbohydrates between control and experiment); (**E**) changes in the content of MDA in the biomass of Spirulina grown in media supplemented with metals; (**F**) modification of the antioxidant activity of Spirulina extracts grown in media supplemented with metals (*p* < 0.01 for differences between control and experiment, for all parameters, except for the case of aqueous extract in Re system at exponential phase and Re-Cu system at stationary phase), (cultivation days 1, 3, 5, when metal ions were introduced into the cultivation medium; biomass was determined on the sixth day of cultivation).

The addition of metal ions to the cultivation medium, in most cases, affects biomass productivity and biochemical composition in either a positive or negative way. Regardless of the time of addition of metals to the cultivation medium, Spirulina went through a full cycle of growth in a closed system. During the stationary phase of culture growth, the biomass was collected, and biochemical changes in the amount of the major components and antioxidant activity of the biomass were traced, as reflected in Figure 2.

In the systems (Re and Re-Mo), which persisted throughout the entire cultivation cycle, the amount of biomass increased when adding metals at all stages of biomass growth (Figure 2a). Numerically, this increase was rather modest—up to 14.3% compared to the control; however, these differences were statistically significant: *p* < 0.005. 

Although the biological role of rhenium for a living organism is unknown, its addition in different phases of biomass growth had beneficial effects on the amount of accumulated biomass. This may be a hormesis effect. Instead, molybdenum is involved in the metabolism of purine bases, being part of the prosthetic group of flavoprotein enzymes [43]. Thus, in the Re-Mo system, the addition of metal ions at the stationary stage of Spirulina growth stimulated an increase in biomass productivity. In both systems, the protein content in the biomass was also higher compared to the control (Figure 2a). Thus, in the Re system, the addition of metal to the medium on the first and third days of biomass cultivation resulted in an increase in the amount of proteins compared to the control by 21.5 and 17.1%, respectively. With the addition of rhenium on the fifth day of biomass growth, the increase was more modest—only by 7.1% (*p* = 0.016). In the Re-Mo system, the amount of protein in the metal-loaded biomass was 28.6, 28.7, and 17.4%, respectively (depending on the phase of biomass growth), higher compared to the control.

In the case of the Re-Cu system, the addition of metals in the lag phase led to the death of the Spirulina culture. This is not surprising, since the toxic effect of copper on cyanobacteria was shown in several studies, the amount of 5 mg/L being an inhibitory one. However, according to previously performed studies, both in the monometal systems and in certain combinations of metals, Spirulina culture can survive in the presence of the specified copper concentration [44,45]. In the present study, Spirulina resisted the action of 0.5 mg/L of copper in the system with rhenium when metals were added to the medium on the third and fifth days of biomass cultivation. Moreover, the amount of accumulated biomass was at the level of the control. However, the negative effect on the Spirulina culture was expressed in a significant decrease in protein content. With the addition of metals on the third day of cultivation, the amount of proteins decreased by slightly more than 7% compared to the control (*p* = 0.0076), but remained within the limits acceptable for culture. This effect was much more pronounced when metals were added on the fifth day of cultivation, when the amount of protein in the biomass decreased by almost a third compared to the control.

Even in the Re-Cu-Mo system, the Spirulina culture did not survive when metals were added on the first day of cultivation. When adding metals in the exponential and stationary phases, the amount of the biomass accumulated at the end of the experiment was lower (statistically significant) compared to the control. The protein content in the biomass was at the level of the control when adding metals in the exponential phase of growth, and reduced by 19.2% in the stationary phase of growth. Thus, at the studied metal concentrations, the negative effects on the Spirulina culture were most likely caused by the presence of copper in the systems.

### 3.3. Effect of Rhenium-Containing Effluents on the Pigment Content of A. platensis 

Primary and secondary photosynthetic pigments are important components of Spirulina biomass, playing important roles in capturing light energy and using it in the synthesis of organic compounds. Spurilina contains a wide range of pigments; among which, phycobiliproteins, carotenoids, and chlorophyll α can be mentioned. Chlorophyll α has the primary photosynthetic role—it captures light. C-phycocyanin and allophycocyanin, which are organized in phycobilisomes, perform the same function. Carotenoids are the most efficient scavengers of singlet oxygen. While performing their main function—the protection of chlorophyll molecules—they also protect biopolymers in the cells of photosynthetic organisms from the destructive effects of light. Derivatives of these pigments are characterized by high antioxidant activity, providing protection to cells from toxic effects, including metals.

Thus, monitoring the content of pigments in Spirulina biomass makes it possible to quickly identify changes occurring in cells under the influence of various stress factors. The results obtained in this regard can be seen in Figure 2b,c.

The most pronounced changes in the case of the analyzed systems occurred in the content of phycobiliproteins (Figure 2b). In the control biomass, the total amount of phycobiliproteins was 11.42% of the dry biomass, including 9.47% of phycocyanin and 1.95% of allophycocyanin. In the experimental variants, the amount of phycobiliproteins was extremely diverse, the values varying between 1.26 and 17.02%. Only in two systems—the Re-system, when the metal was added on the third day, and the Re-Mo system, when metals were added on the first day of biomass growth—the amount of total phycobiliproteins exceeded the control value. In other cases, a significant decrease in the total content of phycobiliproteins was observed. It should be noted that not only the total content, but also the ratio of these two pigments, changed. In the main part of the experimental variants, the amount of allophycocyanin was greater compared to C-phycocyanin, which is atypical for Spirulina.

In the case of the Re-system, when the metal was added in the lag phase, the amount of total phycobilins was 21.62% lower compared to the control (Figure 2b). It is important to note that this decrease occurred due to C-phycocyanin, the content of which decreased by 54.8%, whereas the amount of allophycocyanin increased 2.4 times compared to the control. In this experimental variant, there was a slight decrease in the content of chlorophyll α and an increase in the amount of carotenoids by 25.4% compared to the control (Figure 2c). When Re was added on the third day of cultivation, the amount of total phycobiliproteins increased by 23.7% compared to the control; the increase was ensured by tripling the amount of allophycocyanin, whereas C-phycocyanin was 86.57% of the control value. It should be noted that the amount of other pigments also increased: chlorophyll α by 25.3% and carotenoids by 55.6% compared to the control. The addition of Re at the beginning of the stationary phase caused a decrease in the amount of pigments in the Spirulina biomass. Thus, the total amount of phycobiliproteins decreased by 41% compared to the control, and the ratio between C-phycocyanin and allophycocyanin was disproportionate.

A special situation was observed in the case of the Re-Mo system, when metals were added on the first day of biomass cultivation. The amount of total phycobiliproteins increased by 49% compared to the control. It is important to note that in this variant of the experiment, both the amount of allophycocyanin and C-phycocyanin increased by 152.6% and 27.7%, respectively, compared to the control. The amount of chlorophyll α also increased 1.55 times, and that of carotene 1.9 times. When adding metals at the exponential and stationary stages, the amount of phycobiliproteins changed in a very similar manner. Thus, their quantity decreased by 66.7 and 60.7%, respectively, compared to the control. The amount of C-phycocyanin decreased drastically, by 89.2 and 85.3%, respectively, and the amount of allophycocyanin increased 1.48 and 1.59 times, respectively.

In copper-containing systems, the amount of phycobiliproteins also decreased. In the two-component system, with the addition of metals on the third day of cultivation, the amount of total phycobiliproteins decreased practically two times compared to the control; in particular, C-phycocyanin decreased quantitatively 5 times, whereas the amount of allophycocyanin increased 1.94 times. When adding Re and Cu at the beginning of the stationary phase, the situation was even worse, total phycobiliproteins constituting only 11% of the amount characteristic for the control. However, the amount of chlorophyll and carotenoids in both systems was maintained at a high level, overpassing that of the control. Thus, when rhenium and copper were added on the third day, the amount of carotenoids in the biomass increased by 85.9%, and that of chlorophyll, by 35.1% compared to the control, and on the fifth day, by 30.9 and 45.4%, respectively. The situation in Re-Mo-Cu was very similar to the previously described system.

Analyzing the changes in the amount of pigments in the Spirulina biomass, it can be assumed that maintaining an adequate level of productivity is ensured by the presence of a sufficient amount of chlorophyll α and carotenoids in the biomass. In most of the previously performed studies, the negative influence of copper ions on the content of phycobiliproteins in the biomass of cyanobacteria was attested [44,45,46], which is also confirmed by the data obtained in the present study.

### 3.4. Effect of Rhenium-Containing Effluents on the Content of Carbohydrates and Lipids in A. platensis

The amount of carbohydrates was also affected by the presence of metals in the cultivation medium. The accumulation of carbohydrates in biomass under stress conditions plays a dual role—the formation of carbon reserves and the annihilation of the toxic effects of metal ions on vital molecules (AND, enzymes). In the present study, the response of the Spirulina culture in terms of the amount of carbohydrates was very different and depended both on the chemical composition of the system and the phase when metals were added (Figure 2d). Thus, depending on the studied system, there was an increase in the amount of carbohydrates up to 201% and a decrease up to 10% compared to the control. The addition of rhenium in the monosystem on the first and third days led to an increase in the amount of carbohydrates in the biomass—3.0 and 2.5 times, respectively. When the metal was added on the fifth day, the amount of carbohydrates in the biomass was at the level of the control. In the Re-Mo system, a similar situation was observed; however, the increase in the content of carbohydrates was more modest. Thus, when metals were added on the first day, the amount of carbohydrates in the biomass increased 1.35 times, and on the fifth day—1.16 times (*p* = 0.013). With the addition of metals in the stationary phase of biomass growth, the amount of carbohydrates remained at the level of the control. In the Re-Cu system, the addition of metals on the third and fifth days did not produce significant changes in the amount of carbohydrates in the Spirulina biomass, and in the case of the Re-Mo-Cu system, a moderate increase was observed by 11.3% (*p* = 0.038) and 22.7% (*p* = 0.0046) on the third and fifth days, respectively. 

The amount of lipids in the biomass also changed in the presence of different combinations of metals (Figure 2d). In the Re-system, the content of lipids in the biomass increased with the addition of rhenium in all phases of biomass growth. On the first day of cultivation, the increase was 117.5% compared to the control, on the third day—81.9%, and on the fifth day the increase was much more modest—by 8.9% compared to the control (*p* = 0.035). The same pattern was noticed in the Re-Mo system, in which the lipid content increased by 116.5% when metals were introduced in the lag phase of biomass growth; by 54.2% when added in the exponential phase; and by 19.2% in the stationary phase. In the Re-Cu system, the addition of metals at the exponential stage of cultivation resulted in an increase in the lipid content by 43.5%, and in the stationary phase, this parameter remained at the level of the control. In the Re-Mo-Cu system, the same pattern was manifested, except that the increase in the amount of lipids with the addition of metals at the exponential stage was more modest—by 16.1% (*p* = 0.0098).

One of the most pronounced effects of ROS consists of the oxidative degradation of lipids. Since lipids form the structural basis of biological membranes, the oxidative degradation of lipids has immediate effects on cells, determined by the change in membrane fluidity and permeability, which leads to cell degradation or, at least, to the compromise of biochemical processes that take place on functional membranes. One of the end products of oxidative degradation of lipids is malonic dialdehyde, which is accepted as a recognized marker of oxidative stress.

In all experimental variants (Figure 2e), the amount of MDA was higher compared to the control (*p* < 0.005). The stress to which Spirulina was subjected can be explained in several ways. Thus, in the case of Re-system when rhenium was introduced on the first day of biomass growth, the MDA level increased by 69.2% compared to the control, whereas the content of lipids in the cells increased 118.5% times. This regularity can be regarded as an example of the stimulation of lipid production. With the addition of Re in the exponential phase, the content of MDA increased 3.79 times compared to the control, whereas the amount of lipids increased by 81.9%. This situation should be treated more carefully compared to the previous case; it can be regarded as both moderate stress and stimulation. On the other hand, with the introduction of Re at the stationary stage, the increase in the MDA level by a factor of 1.87 compared to the control, and the preservation of the level of lipids characteristic for Spirulina, indicate a stressful situation in which the process of oxidative biodegradation occurs with greater intensity. In the case of the Re-Mo system, the amount of MDA increased by 158.2; 47.3 and 64.8% depending on the phase of introduction of metals into the medium. The amount of lipids in the biomass also increased. With the addition of metals on the first day, a stimulating effect can be assumed, whereas in the exponential and stationary phases, in addition to these two indicators, there was a strong decrease in the amount of phycobiliproteins, indicating a state of deep stress. In systems containing copper, the level of MDA increase was very pronounced, exceeding its level in the control biomass by a factor of 2.7–5.0. It should be mentioned that the effect was much more pronounced when metals were added at the stationary stage of biomass cultivation.

An increase in the level of MDA in Spirulina cells growing in the presence of copper was observed in numerous experiments, even at concentrations much lower than those used in the present study. Thus, a significant increase in MDA in Spirulina is observed at a copper concentration of 0.1 and even 0.05 mg/L [47,48].

### 3.5. Effect of Rhenium-Containing Effluents on A. platensis Antioxidant Activity

The state of stress caused by the presence of metals in the cultivation medium was also confirmed by a significant increase in the antioxidant activity of the hydric and ethanolic extracts obtained from Spirulina. The increase in % inhibition of the radical cation ABTS^+^˙, depending on the analyzed system, was 44–140% higher compared to the control. The highest values were achieved for extracts from biomass grown in the Re-Cu-Mo system. The accelerated accumulation of hydro- and fat-soluble components in the biomass, which have a pronounced antioxidant activity, can be considered as a crucial event that allows maintaining the viability of the Spirulina culture under conditions of stress caused by metals, especially in copper-containing systems, where the level of oxidative stress was more pronounced.

## 4. Conclusions

The strategy of rhenium recovery from industrial effluents depends on the chemical composition of the effluents. Thus, rhenium recovery from a single metal system or from effluents containing in addition to rhenium molybdenum ions can be performed using Spirulina culture in the lag phase. Rhenium recovery from solutions containing copper could be performed using culture in the stationary phase of growth, and in the case of solutions containing rhenium, copper, and molybdenum, culture in the exponential phase of growth can be used. Rhenium added to Spirulina culture in the lag phase not only accumulates more efficiently, but also has a stimulating effect on Spirulina, increasing the production of biomass, and leading to an increase in the content of protein, carbohydrates, lipids, and pigments in biomass in the absence of pronounced oxidative stress. The presence of copper ions in the effluents provoked a toxic effect on Spirulina, leading to a decrease in biomass productivity, as well as the content of proteins, phycobiliproteins, and carbohydrates. The survival of the biomass in these systems can be explained by the maintenance of a high amount of chlorophyll α and carotenoids in the biomass. *A. platensis* can be regarded as a promising object for mono- and multimetal rhenium-containing effluent treatment.

## Data Availability

Not applicable.
